# Melatonin attenuates cadmium-induced ovulatory dysfunction by suppressing endoplasmic reticulum stress and cell apoptosis

**DOI:** 10.1186/s12958-019-0502-y

**Published:** 2019-07-29

**Authors:** Qingling Yang, Jing Zhu, Xiaoyan Luo, Fangyuan Li, Luping Cong, Yujiao Wang, Yingpu Sun

**Affiliations:** 1 Reproductive Medical Center, First Affiliated Hospital of Zhengzhou University, Zhengzhou, China; 2 Henan Province Key Laboratory for Reproduction and Genetics, Zhengzhou, China

**Keywords:** Cadmium, Melatonin, Ovulation, Endoplasmic reticulum (ER) pathway

## Abstract

**Background:**

Increasing evidence demonstrate that cadmium (Cd) has adverse effects on the mammalian reproductive system. However, the mechanisms underlying the effects of Cd on ovarian function and the strategies to reverse these effects have not been fully elucidated.

**Methods:**

In this study, 60 CD-1 mice were divided into four groups (control, melatonin, Cd, Cd with melatonin). During the treatment for 14 days, body weight was measured every 2 days. After the treatment, ovaries were isolated and weighted to observe the morphological and biological characteristics. Statistical analyses were performed using one-way ANOVA followed by Fisher’s-multiple range test or chi-squared test, A *P* value < 0.05 indicated statistical significance.

**Results:**

We observed that Cd exposure induced ovulatory dysfunction, demonstrated by the reduced number of ovulated oocytes numbers in the Cd group. However, this endoplasmic reticulum (ER) pathway was activated in the Cd-exposed ovaries and the expression of GRP78, ATF4, CHOP, and p-JNK was upregulated, which was reversed by treatment with melatonin. Furthermore, we found that melatonin inhibited Cd-induced activation of cleaved caspase-3, restored the ratio of Bax/Bcl-2, and ultimately decreased the apoptosis of granular cells as detected by TUNEL staining.

**Conclusion:**

Collectively, our findings reveal that melatonin attenuated Cd-induced ovulation dysfunction and cell apoptosis by inhibiting the activation of the ER pathway. Thus, melatonin can be a potential agent to protect mammalian ovaries against Cd toxicity.

## Introduction

Cadmium (Cd) is a ubiquitous environmental contaminant, which cannot be degraded [1]. Exposure to Cd in the general population occurs through the consumption of polluted food or water, cigarette smoking and the inhalation of contaminated air [2]. Occupational exposure also occurs usually due to mining, welding, electroplating, and the manufacture of Cd-containing batteries and pigment [3]. Cd accumulates in many tissues including lung, kidney, pancreas, liver, testes, and ovaries, once absorbed [4, 5].

Mammalian ovaries contain oocytes at different stages of development, and somatic cells of various types. Ovulation is a periodic event, the number of ovulated oocytes strongly associated with female fertility [6]. Increasing evidence demonstrates the reproductive toxicity of Cd in female animals and humans. In rodents, exposure to Cd damaged the structure of the ovary, and caused irregular estrous cycles and abnormal hormone synthesis and follicle development [7, 8]. A recent study also reported the toxicity of Cd on the reproductive system of female birds demonstrated by the ultra-structural changes in the ovarian cells [9]. Cigarette smoking is considered a major source of Cd in humans. Women who smoke showed the shorter menstrual cycles, lower level of estradiol, higher risk of infertility compared with non-smokers [10]. Previous studies suggest that exposure to Cd could induce apoptosis by activating the endoplasmic reticulum (ER) pathway [11]. The accumulation of unfolded proteins in the ER leads to ER stress, and triggers the unfolded protein response (UPR) [12, 13]. The elevated ER stress activates cell apoptosis through (I) the pro-apoptotic transcriptional factor C/EBP homologous protein (CHOP); (II) the apoptosis signal-regulating kinase1 (ASK1)/c-Jun amino terminal kinase (JNK) cascade, and (III) Bax/Bcl-2 [14, 15]. Furthermore, a study on obese mice demonstrated that ER stress is associated with ovulation disorders and deteriorated oocyte quality [16]. However, it is not known if ER stress response is involved in Cd-induced ovulatory dysfunction.

Melatonin (N-acetyl-5-methoxytryptamine), a hormone secreted mainly by vertebrate pineal gland. Peripheral tissues such as retina, gut, ovary, and placenta can also produce this indoleamine hormone [17–19]. Melatonin modulates oxidative stress [20–22], ER stress [23], inflammation [24], apoptosis [25, 26], and autophagy [27] in different disorders. Recent studies suggest that melatonin is able to attenuate the ischemic brain damage by reversing ER stress [28, 29]. Studied on a mouse model of also demonstrated the protective role of melatonin against the excessive activation of primordial follicles [30, 31]. Melatonin has an antiapoptotic effect not only in somatic cells [32], but also in testicular germ cells [33]. However, little is known about melatonin’s effect on the ovulatory function of Cd-treated female mouse.

The aim of this study was to investigate the detail mechanism of Cd-induced ovary dysfunction, and the possible protective effect of melatonin on the ovary. We found that Cd activated the ER pathway, which ultimately resulted in cell apoptosis in the ovary. These conditions were partly alleviated by melatonin, which suggests the potential clinical application of melatonin in the protection of ovarian function.

## Materials and methods

### Animals experiments

Female CD-1 mice (5-week-old) were purchased from Beijing Vital River Laboratory Animal Technology Co., Ltd. (Beijing, China). All the animals were kept under a temperature- and humidity-controlled condition on a 12- h light/dark cycle. They were allowed to acclimatize for a week before treatment and had ad libitum access to food and water. All the experiments have received the approval of institutional review board and the First Affiliated Hospital of Zhengzhou University.

Sixty CD-1 female mice were randomly assigned to four groups. The mice were administered intraperitoneal injections of the vehicle or, melatonin (25 mg/kg; Sigma, St. Louis, Mo, USA), cadmium chloride [8] (CdCl_2_, 5 mg/kg; Sigma, St. Louis, Mo, USA) with or without melatonin [30] (25 mg/kg) daily for 2 weeks respectively. Melatonin stock solutions were dissolved in 100% ethanol and diluted in saline. The final ethanol concentration in the working solutions did not exceed 0.1%. CdCl_2_ was dissolved in saline (5 mg/kg).

### Ovary isolation and oocyte collection

Ovaries were collected, fixed, and embedded in paraffin. Tissues were serially sectioned (5-um thickness) and stained with hematoxylin and eosin. Images were captured with the Nikon Ni-E microscope. To assess the ovulation function, pregnant mare serum gonadotropin (PMSG, 7.5 I.U.) was intraperitoneally injected to stimulate the ovaries of the mice, followed by injection of 7.5 I.U. human chorionic gonadotropin (hCG) after 48 h to induce super-ovulation. After 12–14 h, the mice were sacrificed and the oviductal ampullae were broken to obtain the cumulus-oocyte complexes (COCs). Subsequently, COCs were pipetted in the in-vitro fertilization (IVF) medium (Vitrolife Sweden AB) containing 5% human serum albumin (Irvine Scientific) and 0.1% hyaluronidase (Sigma) to remove the cumulus cells. The number of ovulated oocytes was counted under a stereoscopic microscope (Nikon SMZ800N, Tokyo, Japan).

### Western blot analysis

At least three ovaries were homogenized in the lysis buffer from the protein extraction kit (Sangon Biotech, Shanghai, China). A protein quantitation kit (Bio-Rad) was used to measure the concentrations of the protein solutions. Briefly, the protein samples were separated by 10% SDS-PAGE gel and transferred onto a PVDF membrane. The membranes were blocked with 5% defatted milk in Tris-buffered saline containing Tween20 (TBST) for 1 h at room temperature. Next the proteins were incubated with primary antibodies- GRP78 (1:1000, Cell Signaling Technology, Inc.), ATF4 (1:1000, Cell Signaling Technology, Inc.), CHOP (1:1000, Cell Signaling Technology, Inc.), phosphor-JNK (1:1000, Cell Signaling Technology, Inc.), Bax (1:1000, Proteintech, Inc.), Bcl-2 (1:1000, Proteintech, Inc.), and cleaved caspase 3 (1:1000, Cell Signaling Technology, Inc.), overnight at 4 °C respectively. Anti-GAPDH monoclonal antibody (1:2000, Proteintech, Inc.) was used as a loading control. After incubation with horseradish peroxidase-conjugated secondary antibodies for 1 h at room temperature, the proteins were detected by enhanced chemiluminescence detection system (Bio-Rad).

### RNA extraction and real-time polymerase chain reaction (PCR)

Total RNA was extracted by using the micropurification kit (Qiagen, Dusseldorf**,** Germany), and the synthesis of cDNA was performed with a reverse transcription kit (Takara, Kusatsu, Japan) according to the manufacturer’s protocol. Real-time PCR was performed using the SYBR Green PCR kit (Qiagen, Dusseldorf**,** Germany) and Quantstudio 12 K Flex (Applied Biosystems). The primers sequences of GRP78, ATF4, s-XBP1, CHOP, GAPDH are shown in Table 1. Relative gene expression was calculated basing on the 2^-ΔΔCT^ method. Each sample was measured in triplicate in each experiment.

**Table 1:** Primer sequences

Gene	Forward	Reverse
GRP78	5′-GGTGGGCAAACCAAGACATT-3’	5′-GCCACCACTTCAAAGACACCA-3’
ATF4	5′-GGAATGGCCGGCTATGG-3’	5′-TCCCGGAAAAGGCATCCT-3’
s-XBP1	5′-GGTCTGCTGAGTCCGCAGCA-3’	5′-AGGCTTGGTGTATACATGG-3’
CHOP	5′-TGAAGATGAGCGGGTGGC-3’	5′-TCGTTTCCTGGGGATGAGATA-3’
GAPDH	5′-TGGCAAAGTGGAGATTGTTGCC-3’	5′-AAGATGGTGATGGGCTTCCCG-3’

### Terminal deoxynucleotidyl transferase-mediated dUTP-biotin nick end labeling (TUNEL) assay

To detect the apoptotic rates of the ovarian sections, In Situ Cell Death Detection Kit (Roche, Penzberg, Germany) was purchased to carry out TUNEL analysis based on the manufacturer’s instructions. After incubation in TUNEL reaction mixture for 1 h at 37 °C, the sections were washed twice and counterstained with Hoechst 33342 (10 μg/mL) for 10 min to stain the nuclei. Finally, the sections were mounted on slides and the images were captured under a fluorescence microscope (Nikon Ni-E, Tokyo, Japan).

### Statistical analysis

Data are presented as mean ± SD. All the data were analyzed by one-way ANOVA followed by Fisher’s-multiple range test or chi-squared test, using the SPSS 17.0 Package (SPSS Inc., US). A *p* value < 0.05 indicated statistical significance.

## Results

### Melatonin reverses the ovulatory dysfunction in cd-treated mice

**Fig. 1:**
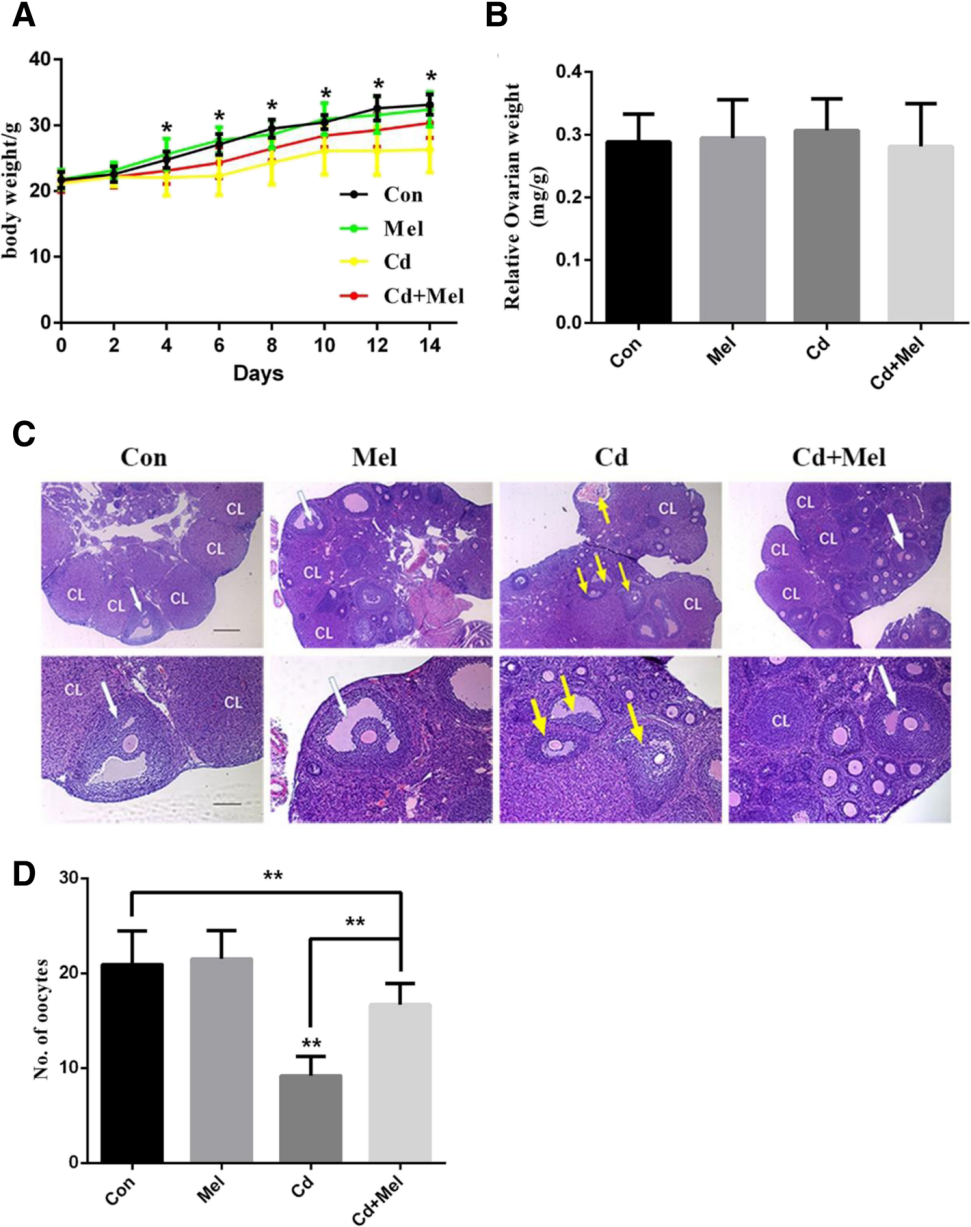
Cd treatment disrupted physiologic functions of ovary. **a** Body weight were conducted every other day during Cd treatment, Cd (5 mg/kg) group are significantly lighter than the other groups from the fourth day of treatment. **b** Comparison of relative ovary weights between the groups. **c** Ovarian morphology by H&E staining. CL, corpus luteum; white arrow, antral follicle; yellow arrow, atretic follicle. The scale bars represent 250 μm (upper panel), 100 μm (lower panel). **d** Number of ovulated oocytes obtained from oviduct. Data are presented as means ± SD. *P < 0.05 and **P < 0.01, compared with the controls

The body weights of the mice were recorded every other day. After treatment for 14 days, the ovaries of each group were harvested, and weighed with an electronic weighing balance. Relative ovary weight was calculated as a proportion of the final body weight before euthanasia, expressed as mg/g body weight. The results showed that the Cd-treated group had significantly less body weight than the control group from the 4th day of the treatment. Melatonin ameliorated the effect of Cd on body weights (Fig. 1a). However, there were no significant differences in the relative ovary weights between the groups (Fig. 1b).

To determine the effects of Cd on ovarian function, we observed the ovarian morphological changes in each group. The ovary consists of follicles at different developmental stages. The formation of antral follicles determined the ovulation in individuals. According to a previous study, the antral follicles had independent antral spaces containing follicular fluid, and the antral follicles were defined as atretic if they contained at least 20 apoptotic granular cells, and a degenerating or apoptotic oocyte [34]. After the injection of the Cd-treated mice with ovulatory gonadotropins, the ovarian histology of the mice showed less antral and more atretic follicles compared to other groups (Fig. 1c), which indicated the ovulatory dysfunction in Cd-exposed ovaries. The addition of melatonin could partially attenuate the degeneration of oocytes. To further evaluate the variation of ovulatory function, we measured the number of ovulated oocytes after the stimulation of super-ovulation. As expected, the number of ovulated oocytes in the oviduct was significantly reduced after Cd treatment, and melatonin considerably restored the impaired oocyte release in the Cd-treated mice (Fig. 1d). These results indicate that intraperitoneal injection of Cd induces ovulatory dysfunction and melatonin could alleviate the damage.

### Melatonin inhibits the ER stress in cd-exposed ovaries

**Fig. 2:**
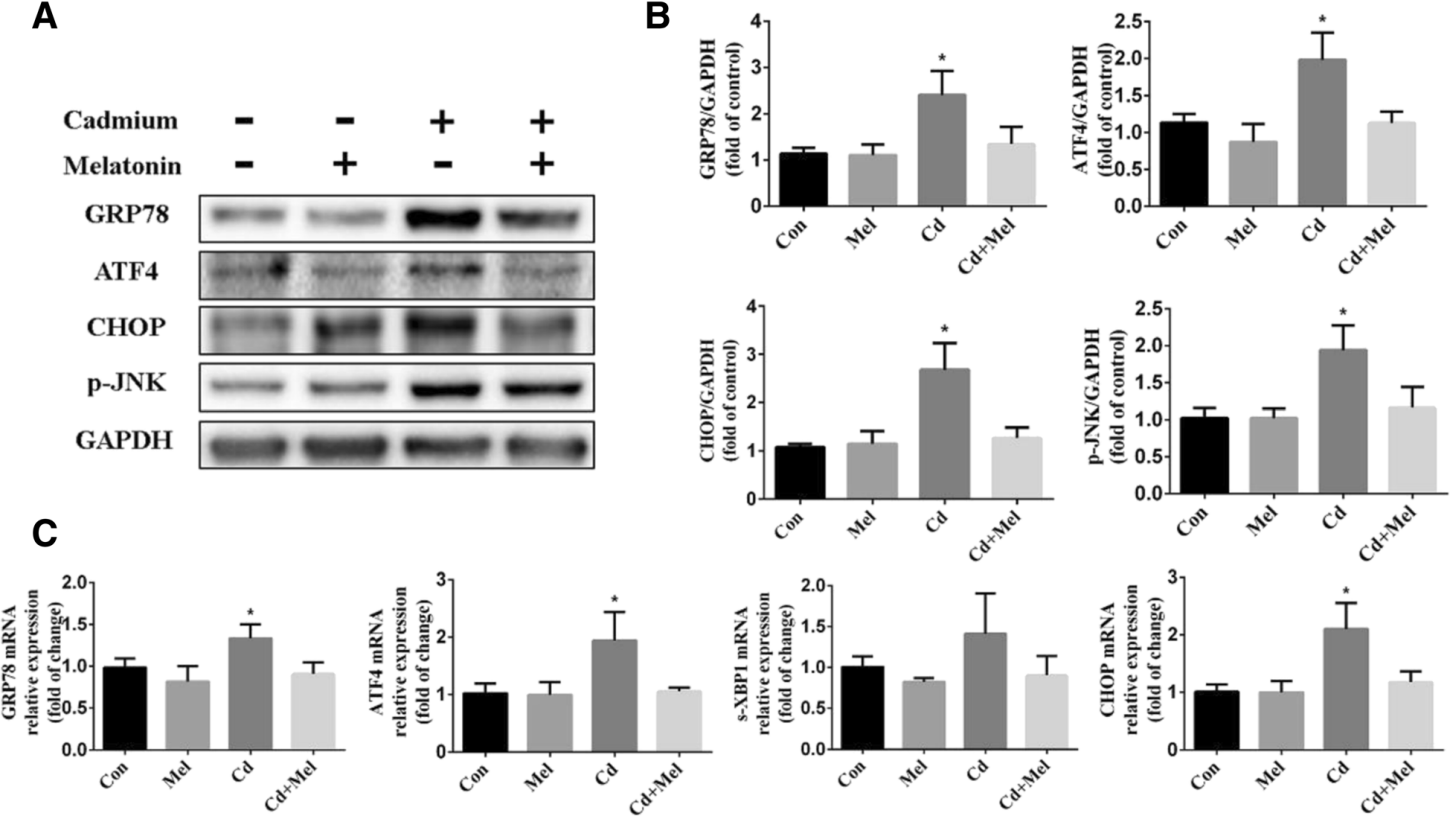
Expression of ER stress-related genes and proteins. **a** Representative western blots of GRP78, ATF4, CHOP and p-JNK expression in 4 groups. GAPDH was used as internal control. **b** Densitometry of western blots was quantified by the ratios to GAPDH. **c** Real-time PCR of ovarian ER stress marker genes (GRP78, ATF4, s-XBP1 and CHOP). Data are presented as means ± SD of at least 3 independent experiments. *P < 0.05, compared with the control group

**Fig. 3:**
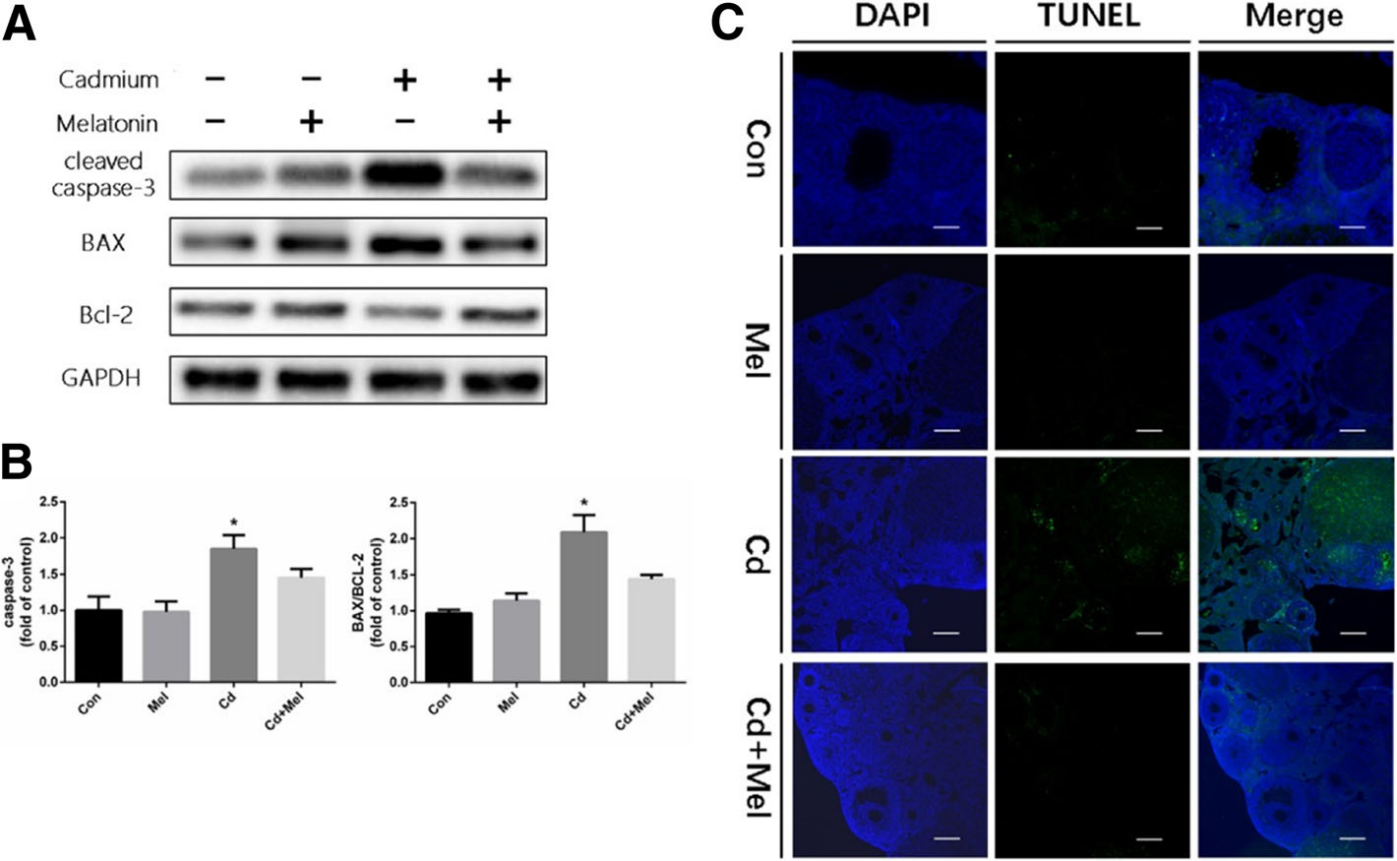
The apoptotic levels in ovaries of each group. **a** Representative western blots of cleaved-caspase 3, BAX and Bcl-2 expression in 4 groups. GAPDH was used as internal control. **b** Activity of cleaved-caspase 3 and the ratio of BAX/Bcl-2. Data are presented as means ± SD of at least 3 independent experiments. *P < 0.05, compared with the control group. **c** TUNEL staining was performed on ovarian sections from the four groups. The green fluorescence represents the TUNEL positive signals. The scale bars represent 250 μm

ER stress has been demonstrated to be a key mechanism underlying Cd-induced cell death. To elucidate the molecular mechanisms underlying the alleviation of ovulation disorders in mice by melatonin, we carried out the western blot assay, and the results showed that the expression of the proteins— GRP78 and ATF4 was significantly upregulated in ovaries of Cd-exposed mice. However, melatonin notably attenuated the Cd-evoked upregulation of GRP78 and ATF4 (Fig. 2a and b). CHOP, a downstream target of ATF4, was analyzed. The results showed that melatonin significantly inhibited Cd-induced upregulation of CHOP (Fig. 2a and b). Phosphorylated JNK (p-JNK), downstream of sXBP-1 also exhibited the similar variation tendency as CHOP (Fig. 2a and b). We also examined the expression of ER stress-related genes (*GRP78, ATF4, spliced XBP-1* transcript, *CHOP*) in the ovaries of different groups (Fig. 3c). The results revealed that Cd exposure significantly increased the mRNA levels of *GRP78, ATF4, CHOP* comparing to the control ovaries, and tended to have higher expression of *sXBP-1*. Notably, administration of melatonin was able to reduce the ER stress in Cd-treated mice comparable to the controls (Fig. 3a). These results suggest that administration of melatonin markedly inhibited ER-stress and its downstream targets, and hence prevented cell apoptosis in the Cd-exposed ovaries. In melatonin-treated group, the ER pathway was not be inhibited, we speculate that the oxidation of tissues is at low levels in normal circumstances, therefore, melatonin does not influence the ER pathway when it was given alone.

### Melatonin inhibits apoptosis in the ovaries

Increased ER stress induced cell apoptosis by activating the caspase pathway; hence, we examined the protein expression of cleaved caspase-3, Bax and Bcl-2 in the ovary by western blotting. Results showed that the protein expression of cleaved caspase-3 indeed substantially increased in Cd-exposed ovaries. Meanwhile, administration of melatonin attenuated the cleaved caspase-3 level indistinguishable from the controls. The Cd group showed a higher level of Bax and a lower level of Bcl-2 protein than the control. Melatonin reversed the negative effects of Cd treatment on the expression of Bax and Bcl-2 proteins. We assessed the Bax/Bcl-2 ratio; results showed that the ratio of Bax/Bcl-2 was considerably higher in the Cd-exposed ovaries than in the ovaried of the mice in the control group. The elevated ratio was reversed by melatonin (Fig. 3a and b). Next, we performed the TUNEL staining to determine apoptosis levels in the ovaries of the mice in the Cd with and the Cd without melatonin groups. TUNEL assay exhibited more positive signals after Cd exposure, and there were more apoptotic granular cells in the developing follicles; the addition of melatonin reduced the number of TUNEL-positive cells in the ovary sections (Fig. 3c), indicating that Cd induced cell apoptosis which could be attenuated by the administration of melatonin.

## Discussion

Cd exposure is associated with female infertility and in vitro fertilization outcomes in humans [35, 36]. In female mice, Cd induces cell apoptosis, and disrupts the normal estrous cycle and hormone synthesis [7, 8]. Therefore, some antiapoptotic compounds have been investigated to evaluate their protective effects against Cd-induced histologic damage. Such compounds include resveratrol [37], quercetin [8], and melatonin [38, 39]. In addition, our previous studies demonstrated the protective effect of melatonin on oocyte quality and the developmental potential of embryo in mice [22]. In the present study, we showed that melatonin partially reversed Cd-evoked pathohistological damage and increased the number of ovulated oocytes. However, the underlying mechanisms were not well elucidated.

ER is sensitive to the disruption of cellular homeostasis. Perturbed ER function, known as “ER stress”, is able to trigger the UPR to restore cellular homeostasis [12, 13]. Earlier studies indicated that Cd-induced cell apoptosis and tissue damage occur through the activation of the ER pathway [33, 40]. In order to establish a link between ER stress and Cd-induced ovulatory dysfunction, we analyzed the expression of related genes and proteins. The mRNA level of sliced *XBP-1*—an important downstream molecule of the IRE1 pathway, was also increased in the ovaries of Cd-exposed mice. The protein and the mRNA levels of GRP78, ATF4 and CHOP were significantly upregulated, indicating the activation of the PERK pathway. These results were consistent with previous findings that Cd affects the reproductive system by elevating the level of ER stress in chicken ovary [9]. Another study also revealed that melatonin could reverse hepatic steatosis through the suppression of ER stress [41]. A recent study reported that melatonin prevented Cd-induced testicular germ cell death via the inhibition of ER signaling [33]. All the results of this study and the findings in previous research indicate that melatonin could partially reverse Cd-induced ER stress.

Excessive ER stress is related to cell apoptosis. CHOP and p-JNK are proapoptotic proteins that could promote the expression of apoptosis-related proteins [14, 15]. The phosphorylation of JNK inhibits the expression of antiapoptotic Bcl-2 protein and promotes the accumulation of active proapoptotic Bax protein [42]. Our results showed a significant upregulation of cleaved caspase 3 and a significantly increased ratio of Bax/Bcl-2 in Cd-treated ovary which corroborates the results of previous studies [8]. In addition, the administration of melatonin significantly downregulated the expression of cleaved caspase 3. The upregulation of Bax and downregulation of Bcl-2 were also partially restored by melatonin. Importantly, melatonin significantly reduced the positive signals of TUNEL staining, indicating its antiapoptotic effect.

## Conclusion

Taken together, our study demonstrated that melatonin can ameliorate Cd-induced ovulatory dysfunction and ovarian injury by suppressing ER stress, suggesting that administration of melatonin has a protective effect against ovary damage caused by Cd exposure. However, additional studies are still needed to investigate the modulatory pathway.

## Data Availability

The data used and analyzed during this study are available from the corresponding author on reasonable request.

## Abbreviations

Cd: Cadmium
COCs: Cumulus-oocyte complexes
ER: Endoplasmic reticulum
hCG: Human chorionic gonadotrophin
Mel: Melatonin
PMSG: Pregnant mare’s serum gonadotrophin
